# Molecular evolutionary process of advanced gastric cancer during sequential chemotherapy detected by circulating tumor DNA

**DOI:** 10.1186/s12967-022-03567-5

**Published:** 2022-08-12

**Authors:** Wenqi Xi, Chenfei Zhou, Fei Xu, Debin Sun, Shengzhou Wang, Yawei Chen, Jun Ji, Tao Ma, Junwei Wu, Chengfang Shangguan, Zhenggang Zhu, Jun Zhang

**Affiliations:** 1 Department of Oncology, Ruijin Hospital, Shanghai Jiao Tong University School of Medicine, No. 197 Ruijin er Road, Shanghai, 200025 China; 2 Department of Oncology, Wuxi Branch of Ruijin Hospital, Shanghai Jiao Tong University School of Medicine, No 197 Zhixian Road, Xinwu District, Wuxi, 214028 China; 3 Shanghai Institute of Digestive Surgery, Ruijin Hospital, Shanghai Jiao Tong University School of Medicine, No. 197 Ruijin er Road, Shanghai, 200025 China; 4 Genecast Biotechnology Co., Ltd, Wuxi City, 214104 Jiangsu China; 5 Department of Radiation Oncology, Ruijin Hospital, Shanghai Jiao Tong University School of Medicine, No. 197 Ruijin er Road, Shanghai, 200025 China; 6 State Key Laboratory of Oncogenes and Related Genes, Shanghai Jiao Tong University, Shanghai, 200025 China

**Keywords:** Gastric cancer, Sequential chemotherapy, Dynamic ctDNA analysis, Copy number variations, Tumor evolution

## Abstract

**Background:**

Efficacy of conventional sequential chemotherapy paradigm for advanced gastric cancer (AGC) patients has largely plateaued. Dynamic molecular changes during and after sequential chemotherapy have not been fully delineated. We aimed to profile the molecular evolutionary process of AGC patients during sequential chemotherapy by next generation sequencing (NGS) of plasma circulating tumor DNA (ctDNA).

**Methods:**

A total of 30 chemo-naïve patients who were diagnosed with unresectable advanced or metastatic stomach adenocarcinoma were enrolled. All patients received sequential chemotherapy regimens following the clinical guideline. One hundred and eight serial peripheral blood samples were collected at baseline, radiographical assessment and disease progression. Plasma ctDNA was isolated and a customized NGS panel was used to detect the genomic features of ctDNA including single nucleotide variants (SNVs) and gene-level copy number variations (CNVs). KEGG pathway enrichment analysis was performed.

**Results:**

Platinum-based combination chemotherapy was administrated as first-line regimen. Objective response rate was 50% (15/30). Patients with higher baseline values of copy number instability (CNI), CNVs and variant allel frequency (VAF) were more sensitive to platinum-based first-line regimens. Tumor mutation burden (TMB), CNI and CNV burden at partial response and stable disease were significantly lower than those at baseline, where at progressive disease they recovered to baseline levels. Dynamic change of TMB (ΔTMB) was correlated with progression-free survival of first-line treatment. Fluctuating changes of SNVs and gene-level CNVs could be observed during sequential chemotherapy. Under the pressure of conventional chemotherapy, the number of novel gene-level CNVs were found to be higher than that of novel SNVs. Such novel molecular alterations could be enriched into multiple common oncologic signaling pathways, including EGFR tyrosine kinase inhibitor resistance and platinum drug resistance pathways, where their distributions were found to be highly heterogenous among patients. The impact of subsequent regimens, including paclitaxel-based and irinotecan-based regimens, on the molecular changes driven by first-line therapy was subtle.

**Conclusion:**

Baseline and dynamic changes of genomic features of ctDNA could be biomarkers for predicting response of platinum-based first-line chemotherapy in AGC patients. After treatment with standard chemotherapy regimens, convergent oncologic pathway enrichment was identified, which is yet characterized by inter-patient heterogenous gene-level CNVs.

**Supplementary Information:**

The online version contains supplementary material available at 10.1186/s12967-022-03567-5.

## Introduction

Gastric cancer is one of the leading causes of cancer death in China with 5-year overall survival (OS) that is barely over 30% [1, 2]. The backbone therapeutic for advanced gastric cancer (AGC) patients remains to be multi-drug combination chemotherapy, which has shown efficacy that has largely plateaued [3]. The median progression-free survival (PFS) of AGC patients is about 6–8 months in first-line setting. Despite progress made in understanding mechanisms that explain chemotherapy resistance of AGC using bioinformatics and multi-omics analysis technologies, biomarkers deemed to be able to identify patients who are more likely to respond to combination chemotherapy are surrounded by controversy [4–6].

Options available to AGC patients as subsequent regimens after first-line treatment are limited [7–9], where the efficacy of current treatment patterns is unsatisfactory. Under the pressure of chemotherapy, tumor cells may evolve to adapt to chemotherapy and acquire a resistant phenotype. Fitness trade-offs occur during this process, which could induce vulnerability to some other drugs. Thus, understanding the molecular evolution in place during the treatment may help to optimize the subsequent treatment. Re-biopsy for late-stage patients is normally difficult, and high inter- and intra-tumoral heterogeneity of gastric cancer suggests that genomic analysis based on tissue biopsy may hardly provide results that are representative of the molecular alterations of the tumor.

Analysis of circulating tumor DNA (ctDNA) using next generation sequencing (NGS) offers a mini-invasive method to avoid the intra-tumoral heterogeneity and to dynamically monitor the molecular alterations in place [10–12]. The correlation between the genomic alterations of ctDNA and the clinical efficacy of the systemic treatment of gastric cancer has already been recognized in the literature [13]. Changes of quantitative data including ctDNA concentration, mutant allele fraction, molecular tumor burden index which have been reported by several research groups can provide indications of cancer recurrence, overall survival, response to systemic therapy of gastrointestinal cancer patients [12, 14, 15]. Somatic mutations of specific genes like *TGFBR2*, *RHOA*, *ERBB2* in ctDNA were found as potential biomarkers of the response to immunotherapy or HER2-trageted therapies [16–18]. However, most of these studies focused on genomic alterations of ctDNA at a specific timepoint during treatment. The dynamic molecular changes and their potential clinical meaning during the sequential chemotherapy in AGC have not been fully delineated.

In the present study, peripheral blood samples were serially and prospectively collected from AGC patients who undergone treatments by sequential chemotherapy regimens. Plasma ctDNA were analyzed utilizing a customized NGS panel. Our aims were to identify the correlations between the genomic features of ctDNA and the patients’ outcomes and to profile the molecular evolutionary process of AGC patients during the sequential chemotherapy to further explore the potential therapeutic options available to these patients.

## Results

### Clinical characteristics of enrolled patients

A total of 30 patients (20 males and 10 females) were enrolled in the study. The patients’ clinical characteristics are listed in Table 1. Seventeen cases had hematogenous metastases, including lung, liver, bone, and adrenal gland. Two chemotherapy regimens were administrated as first-line settings, which included platinum-based triplet regimen (Pt-3d; 14 cases) and platinum-based doublet regimen (Pt-2d; 16 cases) [19]. The details of the treatment results are listed in Additional file 8: Table S1. The best objective response rate of the first-line treatment was 50% (15/30). At the end of the follow-up (January 1st, 2021), four patients were still alive. The median overall survival (OS) of these patients was 9.5 months (range: 2.8–27.6) and the median progression-free survival (PFS) of the first-line setting was 4.8 months (range: 1.5–41.7). A total of 108 peripheral blood samples were eligible for ctDNA analysis, and the median sampling times of the patients was 3 (range 2–10).

**Table 1:** Clinical characteristics of 30 AGC patients

Clinical characteristics	Case (n)	Percentage
Gender		
Male	20	66.7
Female	10	33.3
Age		
Median	64.0	
Range	37–77	
Tumor site		
Fundus	6	20.0
Body	10	33.3
Antrum	14	46.7
Hematogenous metastasis		
Yes	17	56.7
No	13	43.3
Regimens		
Pt-3d	14	46.7
FLOT	11	–
DOS	3	–
Pt-2d	16	53.3
SOX	12	–
CAPOX	3	–
FOLFOX	1	–
PTX	5	16.7
Paclitaxel	2	–
PX	2	–
Nab-paclitaxel	1	–
CPT-11	6	20.0
IRIR	4	–
Irinotecan	1	–
FOLFIRI	1	–
Treatment pattern		
Pt-3d to CPT-11	6	20.0
Pt-2d to PTX	5	16.7
ORR of 1st-line		
PR	15	50.0
SD	10	33.3
PD	5	16.7

*AGC* advanced gastric cancer

### Baseline genomic features of ctDNA correlated with treatment response of first-line regimens

**Fig.1:**
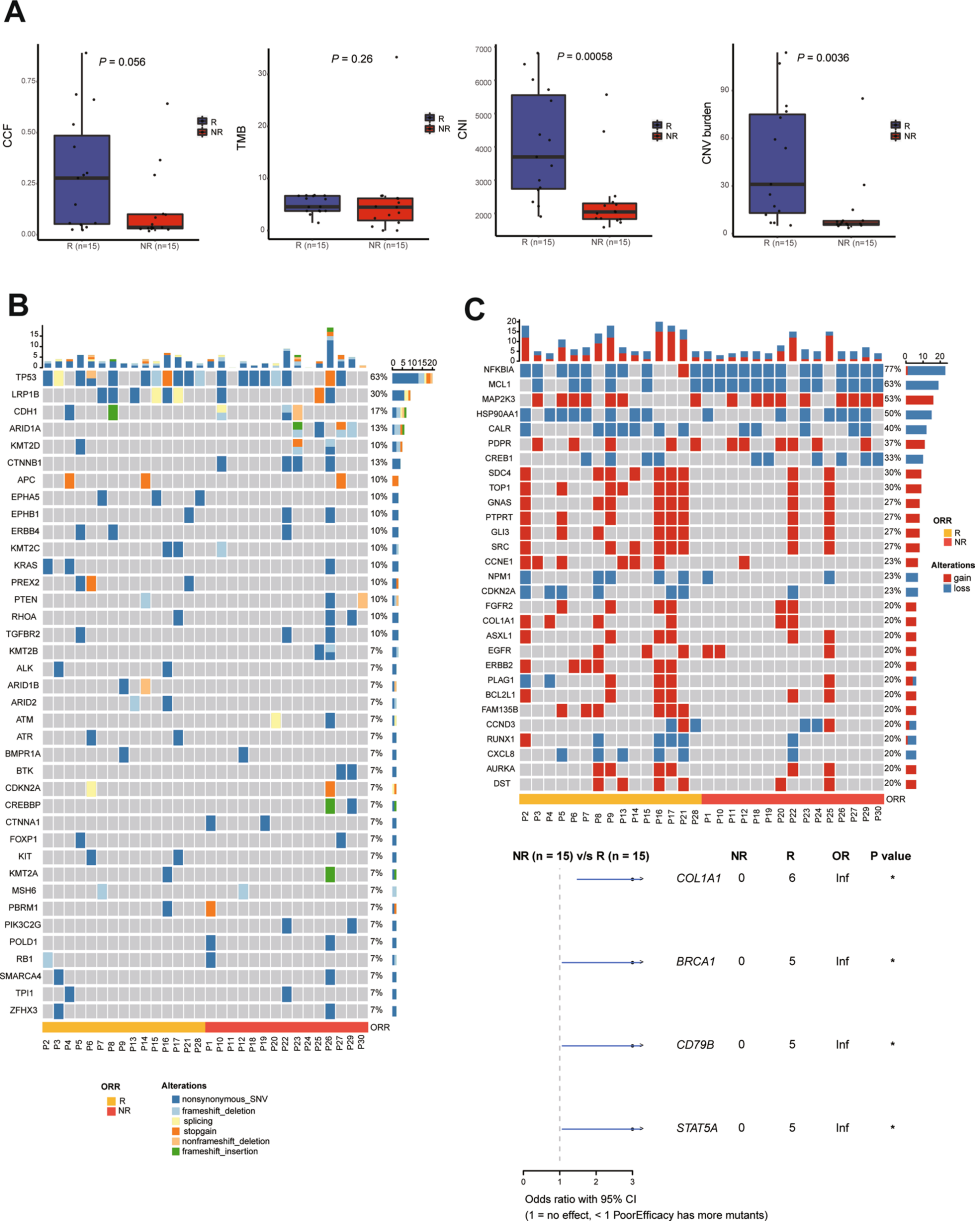
Baseline genomic features of ctDNA correlated with treatment response of first-line regimens. **A**: values of CCF, TMB, CNI and CNV burden in R and NR groups. **B**: landscape of baseline SNVs in all patients. **C**: landscape of baseline CNVs in all patients and four genes correlated with R group

Patients were stratified into R (responsive) and NR (non-responsive) groups by their best objective response to first-line treatment. The clinical characteristics of the two groups were not significantly different (Additional file 8: Table S2). At baseline, compared to NR group, R group showed significantly higher copy number instability (CNI) value (4070.3 ± 1648.7 vs 2406.2 ± 1107.3; *P* = 0.00058) and copy number variation (CNV) burden (45 ± 37 vs 13 ± 21, *P* = 0.0036), whereas values of ctDNA content fraction (CCF) and tumor mutation burden (TMB) were not found to be significantly different (Fig. 1A). Mean variant allele frequency (meanVAF) and maximum variant allele frequency (maxVAF) were also higher in R group (Additional file 1: Figure S1A). The best cut-off value of CNI to stratify R and NR groups was 2339.33 (Additional file 1: Figure S1B). Patients with CNI-high showed similar PFS (*P* = 0.78) and OS (*P* = 0.89) to those of patients with CNI-low (Additional file 1: Figure S1C).

At baseline, top 10 somatic single nucleotide variants (SNVs) were observed in *TP53* (63%), *LRP1B* (30%), *CDH1* (17%), *ARID1A* (13%), *CTNNB1* (13%), *KMT2D* (10%), *APC* (10%), *EPHA5* (10%), *EPHB1* (10%) and *ERBB4* (10%). The distributions of SNVs in R and NR groups were similar (Fig. 1B). The top 10 gene-level CNVs were observed in *NFKBIA* (77%), *MCL1* (63%), *MAP2K3* (53%), *HSP90AA1* (50%), *CALR* (40%), *PDPR* (37%), *CREB1* (33%), *SDC4* (30%), *TOP1* (30%) and *GNAS* (27%). CNVs in R group were more frequent than those in NR group (Fig. 1C). Copy number gain in *COL1A1*, *BRCA1*, *CD79B* and *STAT5A* was only found in R group and was significantly correlated with treatment response of first-line therapy (Fig. 1C). The summarized molecular information on SNV and CNV was shown in Additional file 9: Table S3 and Additional file 10: Table S4, respectively.

### Dynamic change of genomic alterations in ctDNA reflected clinical efficacy during chemotherapy

**Fig.2:**
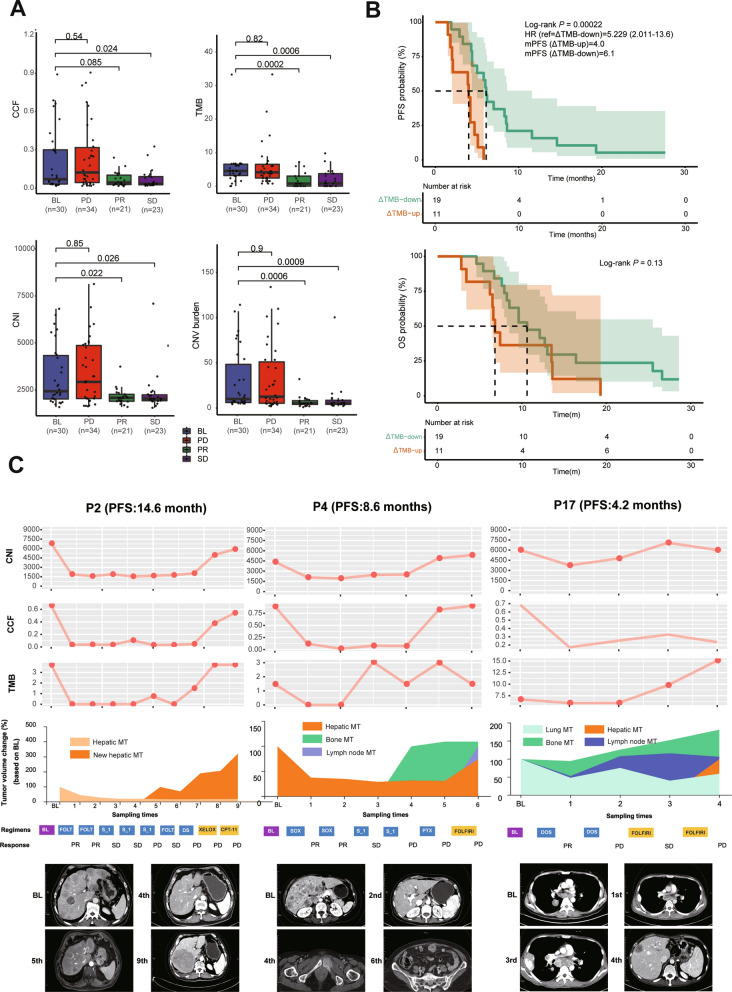
Dynamic change of genomic features in ctDNA correlated with clinical efficacy during chemotherapy. **A**: values of CCF, TMB, CNI and CNV burden at baseline (BL), progressive disease (PD) and during treatment (PR, SD). **B**: correlation between ΔTMB of ctDNA and patients’ outcomes of first-line therapy. **C**: dynamic changes of genomic features in ctDNA and tumor volumes in three typical cases

Values of TMB, CNI and CNV burden at partial response (PR) and stable disease (SD) were significantly lower than those samples at baseline (BL), whereas at progressive disease (PD) they were similar to BL values. The value of CCF at SD was also lower than that at BL, whereas no significant difference was observed between PR and BL (Fig. 2A). Values of meanVAF and maxVAF were not significant different during the treatment (Additional file 2: Figure S2A).

The correlation between early change of genomic features (i.e., delta values between 2nd and 1st sampling points) and the outcome of patients was analyzed. The cut-off value of ΔTMB was defined as −1, where 19 patients were defined as ΔTMB-down (ΔTMB < −1) and 11 patients were defined as ΔTMB-up (ΔTMB > −1). PFS of ΔTMB-down patients was longer than that of ΔTMB-up patients (6.1 months vs. 4.0 months; *P* < 0.001), where OS was similar between two groups (*P* = 0.13; Fig. 2B). For other genomic features, no correlation with PFS was identified (Additional file 3: Figure S3).

The dynamic changes of genomic features in ctDNA and tumor volumes are described in three typical cases (Fig. 2C). The values of CNI, CCF and TMB all decreased after the initial response to the first-line treatment and were kept at low level during the disease control. After disease progression, CNI, CCF and TMB continued to increase during the subsequent treatment.

### Fluctuating changes of genomic alterations during sequential chemotherapy of AGC

**Fig.3:**
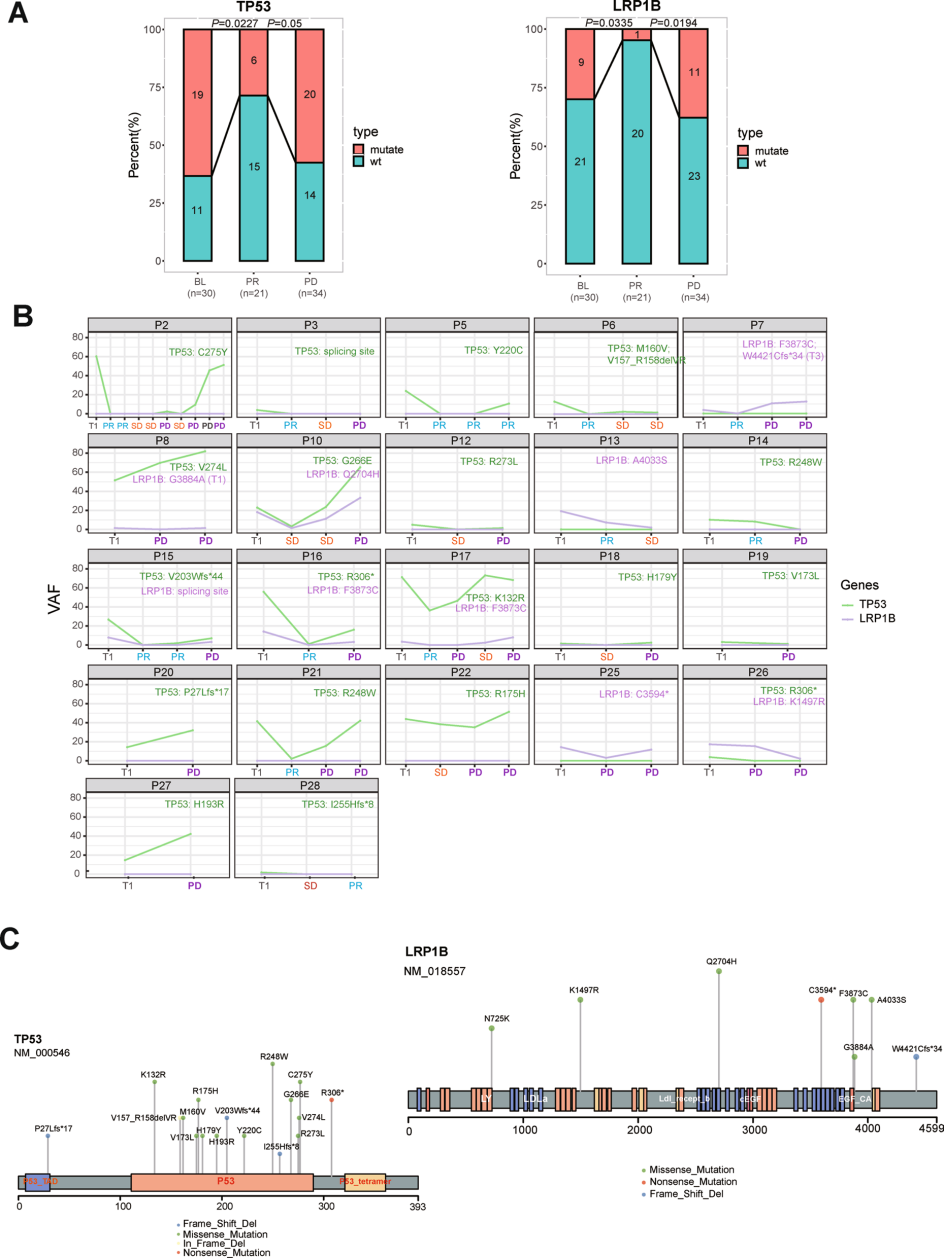
Fluctuating changes of genomic alterations during sequential chemotherapy. **A**: changes of percentages of *TP53* and *LRP1B* mutations during treatment. **B**: fluctuating changes of *TP53* and *LRP1B* frequency during treatment. **C**: involved mutation sites of *TP53* and *LRP1B*

Fluctuating changes could be observed once comparisons were made in SNVs and CNVs landscape patterns of 30 patients. Some SNVs (Additional file 2: Figure S2B) and CNVs (Additional file 2: Figure S2C) were undetectable at PR and SD, which were recovered at PD. *TP53* and *LRP1B* were top two genes with somatic mutations. The percentages of detectable *TP53* and *LRP1B* mutations were lower at PR compared to BL, where they increased at PD (Fig. 3A). For an individual patient, the mutation types and sites were the same during the whole course of the treatment (Fig. 3B). However, the sites of point mutations of these two genes were highly heterogenous among the patients (Fig. 3C).

### Enrichment of novel genomic alterations of ctDNA during treatment as revealed by KEGG analysis

**Fig.4:**
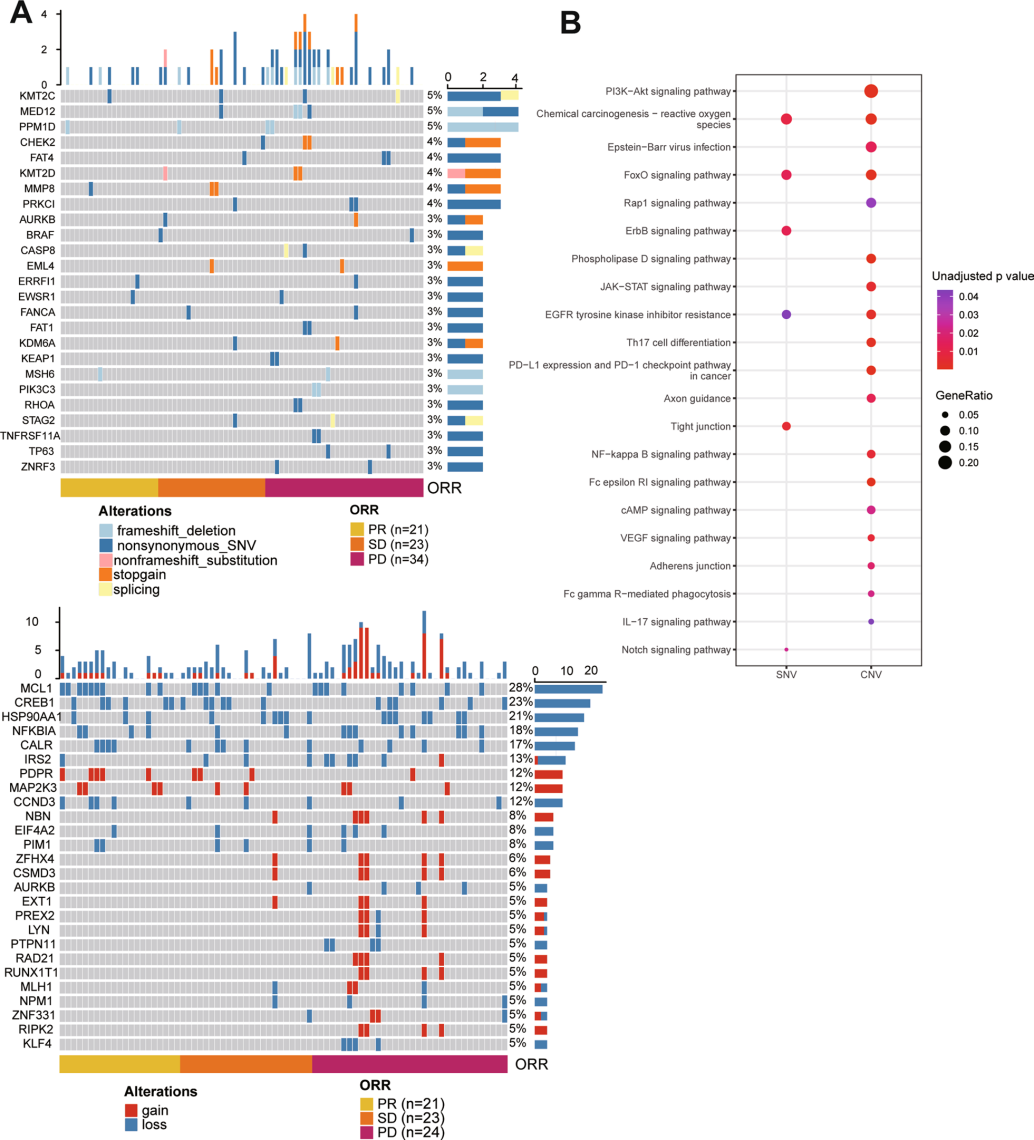
KEGG analysis of novel genomic alterations of ctDNA during treatment. **A**: landscapes of novel SNVs and CNVs in all patients. **B**: signaling pathways enriched by novel SNVs and CNVs

Novel SNVs occurred in 130 genes during the whole course of the treatment. Only 26 genes were found in more than 2 patients. The most frequently novel SNVs were observed in *KMT2C* (5%), *MED12* (5%), *PPM1D* (5%), *CHEK2* (4%), *FAT4* (4%), *KMT2D* (4%), *MMP8* (4%) and *PRKCI* (4%). Novel CNVs occurred in 191 genes, and 109 of them were observed in more than 2 patients. The most frequently novel CNVs were observed in *MCL* (28%), *CREB1* (23%), *HSP90AA1* (21%), *NFKBIA* (18%), *CALR* (17%), *IRS2* (13%), *PDPR* (12%), *MAP2K3* (12%) and *CCND3* (12%). Novel SNVs and CNVs were both more frequent in PD samples (Fig. 4A). KEGG analysis revealed more tumor related signaling pathways could be enriched by novel gene-level CNVs comparing with novel SNV. PI3K-Akt signaling pathway, chemical carcinogenesis-reactive oxygen species, Epstein-Barr virus infection and FoxO signaling pathway were the top enriched tumor related pathways (Fig. 4B). Pathways including platinum drug resistance and PD-L1 expression and PD-1 checkpoint pathway in cancer were also enriched.

### Impact of subsequent chemotherapy on genomic alterations of ctDNA driven by first-line treatment

**Fig.5:**
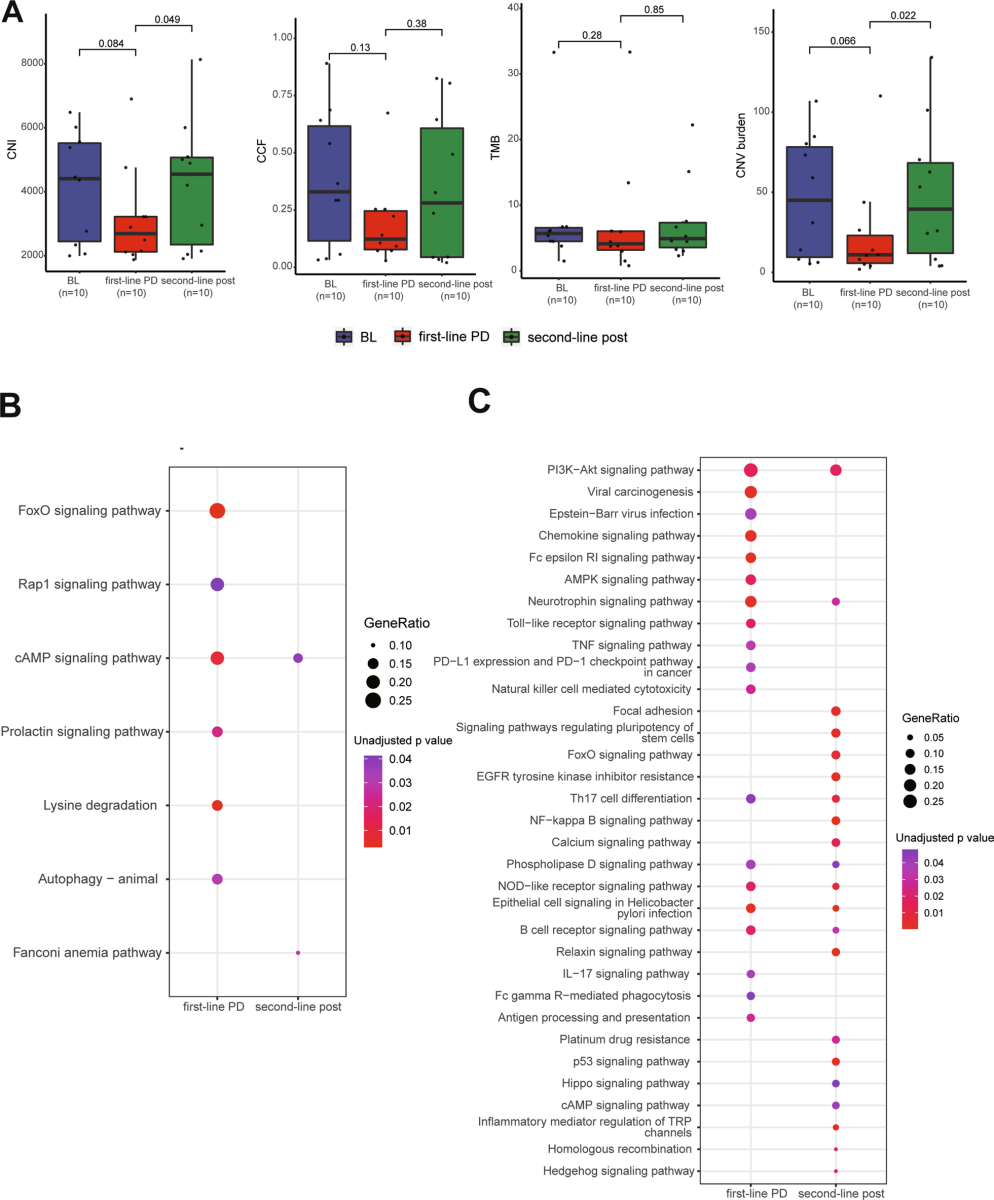
Impact of subsequent chemotherapy on genomic alterations of ctDNA. **A**: values of CNI, CCF, TMB and CNV burden at BL, first-line PD and second-line posttreatment. **B**: signaling pathways enriched by novel SNVs and CNVs at first-line PD and second-line posttreatment

Peripheral blood samples collected from 10 patients during treatments of subsequent regimens were analyzed. Both CNI and CNV burden values increased at second-line post-treatment relative to baseline and first-line PD. CCF and TMB values were not significantly different at these three time points (Fig. 5A). The number of genes with novel CNVs at second-line post-treatment was more than that at first-line PD (186 vs. 45). KEGG analysis of novel SNVs at second-line post-treatment showed that cAMP signaling pathway and Fanconi anemia pathway were the top enriched tumor related pathway (Fig. 5B). Multiple common signaling pathways enriched by novel gene-level CNVs between first-line PD and second-line post-treatment could be identified. NF-kappa B signaling pathway, platinum drug resistance pathway and FoxO signaling pathway could be enriched by novel CNVs at second-line post-treatment rather than at first-line PD (Fig. 5C).

## Discussion

In the present study, both baseline and dynamic changes of genomic features in ctDNA were found to be correlated with the treatment efficacy of first-line chemotherapy for AGC patients. Fluctuating changes in SNVs and CNVs could be observed during sequential chemotherapy, where the impact of the subsequent regimens on the molecular changes driven by first-line therapy was determined to be subtle. Under the pressure of conventional chemotherapy, novel SNVs and CNVs could be enriched into multiple common oncologic signaling pathways, where their distributions were established to be highly heterogenous among patients.

The correlation between the genomic features of ctDNA and the chemotherapy efficacy in AGC patients has been analyzed in previous studies, where most of the studies were focused on reporting the data of somatic mutations, including number of gene mutations and VAF [12, 14, 20]. Recent more detailed analyses of ctDNA revealed that the molecular changes correlated with ant-HER2 targeted therapy and anti-PD-1 immunotherapy [15, 16, 18]. Nevertheless, ctDNA analysis during conventional sequential chemotherapy—which is still the standard treatment pattern for most AGC patients in China—have not been fully explored.

We found that AGC patients who had higher baseline values of CNI and CNV burden in ctDNA were more likely to respond to platinum-based chemotherapy. Somatic CNVs were correlated with chromosome instability, which resulted from defects in mitosis and pre-mitotic replication stress [21]. In AGC patients, it has been reported that high level chromosome instability was associated with sensitivity to platinum-based chemotherapy [22]. In colorectal cancer patients, tumors with increased copy number alterations also indicated sensitivity to chemotherapy plus bevacizumab [23]. Also, chromosome instability has been found to be associated with intrinsic resistance to taxanes [24]. In our study, we found that both CNI and CNV burden in ctDNA recovered to their baseline levels at treatment failure of first-line regimens. The responses to paclitaxel-based and irinotecan-based regimens as second-line treatments were both found to be poor in our cohort. This was in line with the findings of previous randomized controlled trials [9]. Our findings suggest that patients with high CNI and high CNV burden may not benefit from the standard pattern of subsequent chemotherapy of AGC.

Even though the values of CNI and CNV burden at disease progression were similar to those at baseline, treatment resistance still occurred. Novel SNVs and CNVs were observed in ctDNA at PD. Compared with somatic mutations, platinum-based chemotherapy could cause more gene-level CNVs in ctDNA. A pan-cancer study demonstrated the existence of a positive linear influence of CNVs on the expression of most of the genes, which was indicative of the presence of a direct effect of CNVs on gene transcriptional level [25]. Furthermore, gene-level CNVs are considered as common and important molecular alterations in tumors and are usually the upstream changes that trigger the cascade of signaling chaos [26]. In our study, KEGG analysis of novel CNVs showed that several oncologic and treatment-resistance pathways, including EGFR tyrosine kinase inhibitor resistance and platinum drug resistance, were enriched. These novel genomic alterations could be attributed to resistance to the first-line chemotherapy.

Tumor evolutionary theory provides an alternative strategy to optimize the current treatment paradigm of cancer [27]. Spontaneous fitness trade-offs are the cost of evolution during tumor adaptation to the drastic external pressure of chemotherapy. These fitness trade-offs lead to the appearance of vulnerability in tumor cells to specific subsequent therapies [28–30]. This phenomenon creates an opportunity for oncologists to anticipate tumor evolutionary process under the pressure of certain drugs and design an optimal subsequent regimen to prolong disease control [31]. However, although we found that genes with novel SNVs and CNVs could be identified during and after the whole course of the treatment, the corresponding distributions among AGC patients were highly heterogeneous.

Molecular matched therapy has been performed based on molecular alterations detected in tumors. However, the benefit of this strategy in subsequent treatment of gastric cancer is uncertain. SHIVA trial did not show a significant benefit, and the absolute time of survival extension in VIKTORY trial was reported to be only about 2 months [32, 33]. In our study, the genes involved in relevant signaling pathways were found to be highly heterogeneous among patients. It has been concluded that while common aberrant pathways might result in treatment failure, inter-patient heterogeneity and signaling crosstalk could lead to inefficacy of single-targeting molecular agents [34]. Thus, a multi-targeting strategy should be employed in subsequent regimens of AGC patients. In EPOC1706 trial, lenvatinib plus pembrolizumab in the first-line or the second-line setting of ACG patients showed an objective response rate of 69%, which was much higher than that of previously-employed standard second-line chemotherapy of AGC patients [35]. REGONIVO trial also showed a high response rate for the treatment of refractory AGC patients with regorafenib plus nivolumab [36]. The synergistic effects in place between lenvatinib/regorafenib and immune checkpoint inhibitors such as pembrolizumab and nivolumab, together with the fact that both lenvatinib and regorafenib are multi-targeting tyrosine kinase inhibitors of VEGFR, FGFR, PDGFR, KIT and RET, should both account for such enhanced clinical efficacy[37, 38].

Tumor cells can adapt to external treatment pressure by acquisition of new genomic or epigenomic changes [39]. This means that the dynamics of tumor evolution should also be considered in devising therapeutic patterns for cancer. In a study on the genomic landscape of relapsed acute lymphoblastic leukemia, the prevalence of relapse-specific somatic alterations in patients showing very early relapse (< 9 months from diagnosis) was found to be significantly less than that in the patients who exhibited an early or late relapse [40]. The median PFS of AGC patients in the first-line treatment is normally 6–8 months. Despite being for different kinds of tumors, these results suggest that gastric cancer cells might not have long enough time to acquire new driver somatic alterations under treatment pressure. In this context, Li et al. have also reported quite similar CNV profiles in paired tissue samples collected before and after neoadjuvant chemotherapy from gastric cancer patients who had no response [41].

The present study bears certain limitations. First, the fact that only 30 patients were enrolled in our study made the sample size relatively small. Nevertheless, analysis of 108 ctDNA samples could present a representative view over the dynamic molecular changes during the treatment. Second, analysis of subsequent therapy was only performed in 10 patients. The fact that the available information found in the literature regarding the dynamic molecular changes during sequential chemotherapy of AGC patients is limited draws attention to the importance of the results of our study. Third, the analysis of SNVs and gene-level CNVs in our study was not accompanied by the examination of the possible correlations between them and the gene expression. Thus, it is our aim to investigate the transcriptional data in future studies using a larger sample size.

In summary, baseline and dynamic changes of genomic features of ctDNA could be biomarkers to predict treatment efficacy of platinum-based first-line chemotherapy in AGC patients. After treatment with standard chemotherapy regimens, convergent oncologic pathway enrichment yet characterized by inter-patient heterogenous gene-level CNVs was identified, which provided valuable information for the optimization of the sequential treatment of AGC patients.

## Materials and methods

### Patients and samples

Peripheral blood samples were collected from gastric cancer patients who were treated in Department of Oncology, Ruijin Hospital and met the following criteria: (1) age over 18 years; (2) histologically confirmed HER2 negative stomach adenocarcinoma; (3) treatment naïve locally advanced, recurrent or metastasis disease; (4) adequate performance status and organ function to receive standard chemotherapy. Written informed consent was provided by all patients before collecting the peripheral blood samples. The protocol was approved by ethics committee of Ruijin Hospital, Shanghai Jiao Tong University School of Medicine, Shanghai, People’s Republic of China.

Blood samples were collected at times of baseline, efficacy assessment and disease progression. Samples collected at treatment failure of one regimen were considered as baseline samples for the regimen that followed. Regimens with at least two samples during treatment were eligible for further analysis. Treatment efficacy was assessed by radiographic examination (contrast enhanced CT or MRI) following the routine clinical protocols every 8–9 weeks. Objective response was assessed according to the RECIST criteria (v1.1). Clinicopathological information of all the patients was recorded, and PFS and OS were followed up. The best objective response during first-line treatment was used to stratify patients into R and NR groups. During the whole course of treatment, treatment response at each sampling timepoint was compared with last timepoint according to RECIST criteria (v1.1). Tumor response of each timepoint was listed in Additional file 11: Table S5.

### DNA extraction, sequencing and data processing

Plasma and blood cells were separated by centrifugation at 1600 g for 10 min. Cell-free DNA (cfDNA) was isolated from plasma using MagMAX^™^ Cell-Free DNA Isolation Kit (Thermo Fisher Scientific, Waltham, MA, USA). Blood cell DNA was extracted from blood cells with TIANamp Blood DNA Kit (TIANGEN, Beijing, China). The concentration of DNA was measured by Qubit^®^ dsDNA HS Assay Kit (Thermo Fisher Scientific, Waltham, MA, USA), and the quality of DNA was assessed by Agilent 2100 BioAnalyzer (Agilent, USA). Genomic DNA was sheared into 150–200 base-pair (bp) fragments with Covaris M220 Focused-ultrasonicator Instrument (Covaris, Massachusetts, USA). Fragmented DNA was used to construct the library using KAPA Hyper Preparation Kit (Kapa Biosystems, USA) according to the manufacturer’s instruction. DNA was hybridized to one in-house panel (Genecast, Wuxi, China) covering 2.43 Mb of the human genome with 1632 genes (Additional file 12: Table S6). The captured library was sequenced on Illumina NovaSeq 6000 according to the manufacturer’s instruction, producing paired-end reads with the length of each end as 150 bp.

Preliminary sequencing results in BCL format were converted to FASTQ files using bcl2fastq (v2.20.0). FASTQ format reads were processed using Trimmomatic (v0.39) [42] for adapter trimming and low-quality reads filtering. Processed reads were mapped to the reference genome (hg19) using BWA (0.7.17) [43]. Mapped results were sorted and marked for duplications using Picard toolkit (version 2.1.0, https://broadinstitute.github.io/picard/), and then were realigned using GATK (version 3.7) [44]. The quality control data were shown in Additional file 13: Table S7. The average sequencing depth of CF and BC samples was > 1000 × and > 200 × , respectively. The distribution of fragment sizes for each sample is shown in Additional file 4: Figure S4.

### Somatic SNV calling

SNVs were called via VarDict (v1.5.1) and FreeBayes (v1.2.0) from processed mapping results in pair of tumor and control samples, respectively. The candidate somatic mutations were annotated with ANNOVAR (2015Jun17) [45] and then were filtered by ExAC (http://exac.broadinstitute.org), gnomAD (http://gnomad-sg.org/), COSMIC (https://cancer.sanger.ac.uk/cosmic), dbSNP (https://www.ncbi.nlm.nih.gov/snp/) databases. Nonsynonymous mutations among the exonic and splicing regions were kept for the final somatic mutation data set (Additional file 5: Figure S5). The filter parameters of SNV include the following criteria: (1) exclude mutations located in intergenic regions or intronic regions. (2) exclude blacklisted mutations. (3) exclude mutations with sequencing depth  < 120. (4) exclude mutations with allele frequency  < 1%. (5) exclude mutations with reads  < 5. (6) exclude synonymous mutations. (7) exclude allele frequency  ≥ 0.002 in the Exome Aggregation Consortum (ExAC) database. (8) exclude mutations also present in blood control samples and the frequency in tumor sample is less than five times frequency in blood control sample (Additional file 14: Table S8).

### Somatic CNV calling

Taking blood cell sample as the control, somatic CNVs were called from plasma sample for each gene included in the captured panel using CNVkit (0.9.5.dev0) with batch mode [46]. In the process of calling, biases of read depth, GC content and repetitive sequence were removed through normalization (Additional file 6: Figure S6). The filtering criterion of CNV is the number of capture region more than 5, and the threshold of gain and loss are copy ratio  > 2.5 and copy ratio  < 1.5, respectively. The CNV value was defined as log2 transformed ratio of normalized read depth on each gene between plasma and blood cell samples (Additional file 15: Table S9).

### Calculation of tumor mutation burden (TMB)

TMB were determined by the nonsynonymous somatic mutations among the exonic and splicing regions. Alterations likely or known to be bona fide oncogenic drivers were excluded (Additional file 7: Figure S7). TMB per megabase were calculated with the total number of mutations divided by the total bases of the target panel with  ≥ 500 bp coverage.

### Definition of meanVAF and maxVAF

The maxVAF value equals to the maximum VAF of all somatic mutations in each sample. The meanVAF value was defined as the mean value of all mutations’ VAFs in each sample.

### Calculation of CNV burden

CNV burden is equal to the total number of CNVs in each sample. Considering the two types of CNVs, the CNV burden was divided into gain and loss. The total CNV of gain and total CNV of loss were defined as the CNV gain burden and CNV loss burden, respectively.

### Estimation of ctDNA content fraction (CCF)

CCF of plasma samples was estimated by a maximum likelihood model based on SNVs and CNVs in the paired blood cell and plasma samples. Somatic and germline SNPs that met the following criteria were used to build the model: (1) with a minimum depth of 50 × in the paired samples; (2) not on genes with high polymorphism; (3) no InDels in the 50 bp upstream or downstream regions; (4) not in a copy number gain region; (5) germline SNPs with significantly different variant allele frequencies (VAFs) in the paired samples, or somatic SNPs with VAFs significantly higher than background noise. These SNPs were defined as informative SNPs and were clustered into multiple groups according to their VAFs, local copy numbers and hypothetic genotypes. The hypothetic genotypes in cfDNA and ctDNA were determined by the VAFs in the paired samples and the copy number in the plasma sample. Each cluster represents a unique ctDNA source. We then calculated the likelihood of observing SNPs under given CCFs in each cluster. By maximizing the likelihood, CCF of each cluster could therefore be estimated. Cluster with the highest CCF was from the main source of ctDNA, and its CCF was the output of the final estimation.

### Estimation of copy number instability (CNI)

After correction for GC content and length of target region using proprietary algorithms for each region, the read counts were transformed into log2 ratios and were converted into Z-score based on Gaussian transformations versus a normal control group (n = 30). The target regions that satisfied the Z-score greater than the 95th percentile plus twice-times absolute standard deviation of the normal control group were retained, and these Z-scores were summed as the CNI score.

### Statistical analysis

For the comparison of continuous variables, we chose Wilcoxon rank sum test. When a continuous variable was transformed into a categorical one, the best cutoff was determined through pROC (1.18.0) package [47]. Comparisons between categorical variables were conducted using Fisher’s exact test. Kaplan–Meier survival curves with log-rank test were generated by survminer (0.4.9) and survival packages (3.3–1). The visualization of gene alteration landscapes was profiled by ComplexHeatmap package (2.12.0) [48]. The KEGG analysis of newly emerged gene alterations was performed on clusterProfiler package (v4.4.4) [49]. We customized 1632 panel genes as background gene sets for pathways enrichment. All analyses were carried on R (4.2.1) program. *P* < 0.05 was considered as statistically significant.

## Supplementary Information

**Additional file 1: Figure S1. **Baseline genomic features of ctDNA and treatment response of first-line therapy. A: values of meanVAF, maxVAF, CNV gain burden and CNV loss burden in R and NR groups. B: cut-off value of CNI and its correlation with treatment response. C: correlation between CNI value and patients’ overall survival.

**Additional file 2: Figure S2. **Dynamic change of genomic features of ctDNA and landscapes of SNVs and CNVs. A: values of meanVAF, maxVAF, CNV gain burden, CNV loss burden at baseline, disease progression and during treatment. B: landscapes of SNVs of all samples. C: landscapes of CNVs of all samples.

**Additional file 3: Figure S3. **Correlation between dynamic changes of genomic features of ctDNA and progression free survival of first-line therapy.

**Additional file 4: Figure S4. **Insert size distribution.

**Additional file 5: Figure S5. **Coverage distribution.

**Additional file 6: Figure S6. **All uniform Manhattan of blood control samples.

**Additional file 7: Figure S7. **All uniform Manhattan of cell-free samples

**Additional file 8: Table S1. **Treatment paradigms of 30 gastric cancer and the corresponding clinical outcomes. **Table S2.** Examination of the differences between the clinical characteristics of patients in NR and R groups at first-line treatment.

**Additional file 9: Table S3. **SNV information.

**Additional file 10: Table S4. **CNV values of all samples.

**Additional file 11: Table S5. **Information of sampling timepoints and treatment response.

**Additional file 12: Table S6. **Gene list of NGS panel.

**Additional file 13: Table S7. **Quality control.

**Additional file 14: Table S8. **SNV landscape of all ctDNA samples.

**Additional file 15: Table S9. **CNV landscape of all ctDNA samples.
