# Supplementary figures and images for: Molecular evolutionary process of advanced gastric cancer during sequential chemotherapy detected by circulating tumor DNA
Source: J Transl Med. 2022 Aug 12;20:365. doi: 10.1186/s12967-022-03567-5 (PMC9373478; doi:10.1186/s12967-022-03567-5)

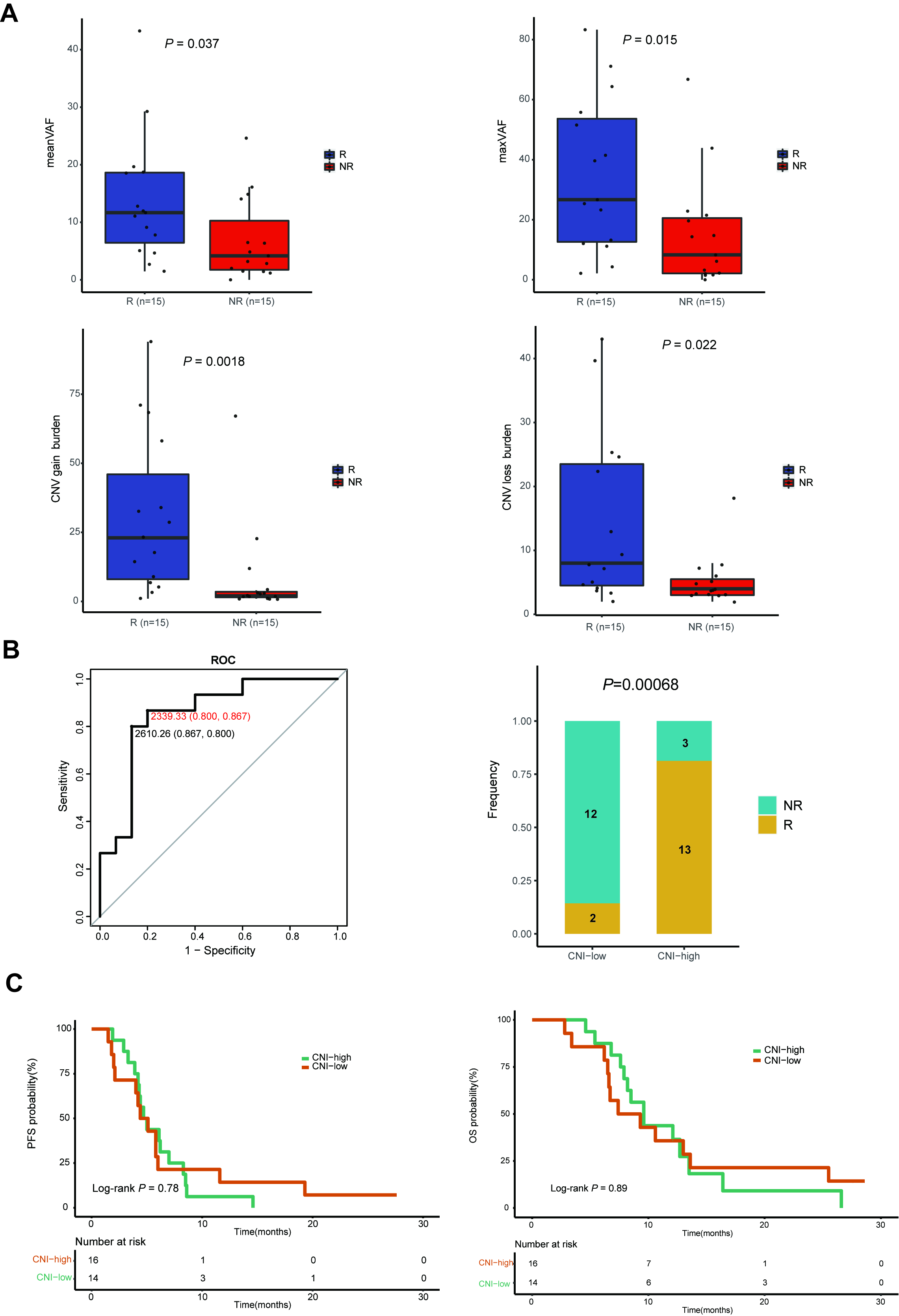

Supplement: Supplementary file 1 — Additional file 1: Figure S1. Baseline genomic features of ctDNA and treatment response of first-line therapy. A: values of meanVAF, maxVAF, CNV gain burden and CNV loss burden in R and NR groups. B: cut-off value of CNI and its correlation with treatment response. C: correlation between CNI value and patients’ overall survival. [file 12967_2022_3567_MOESM1_ESM.tif]

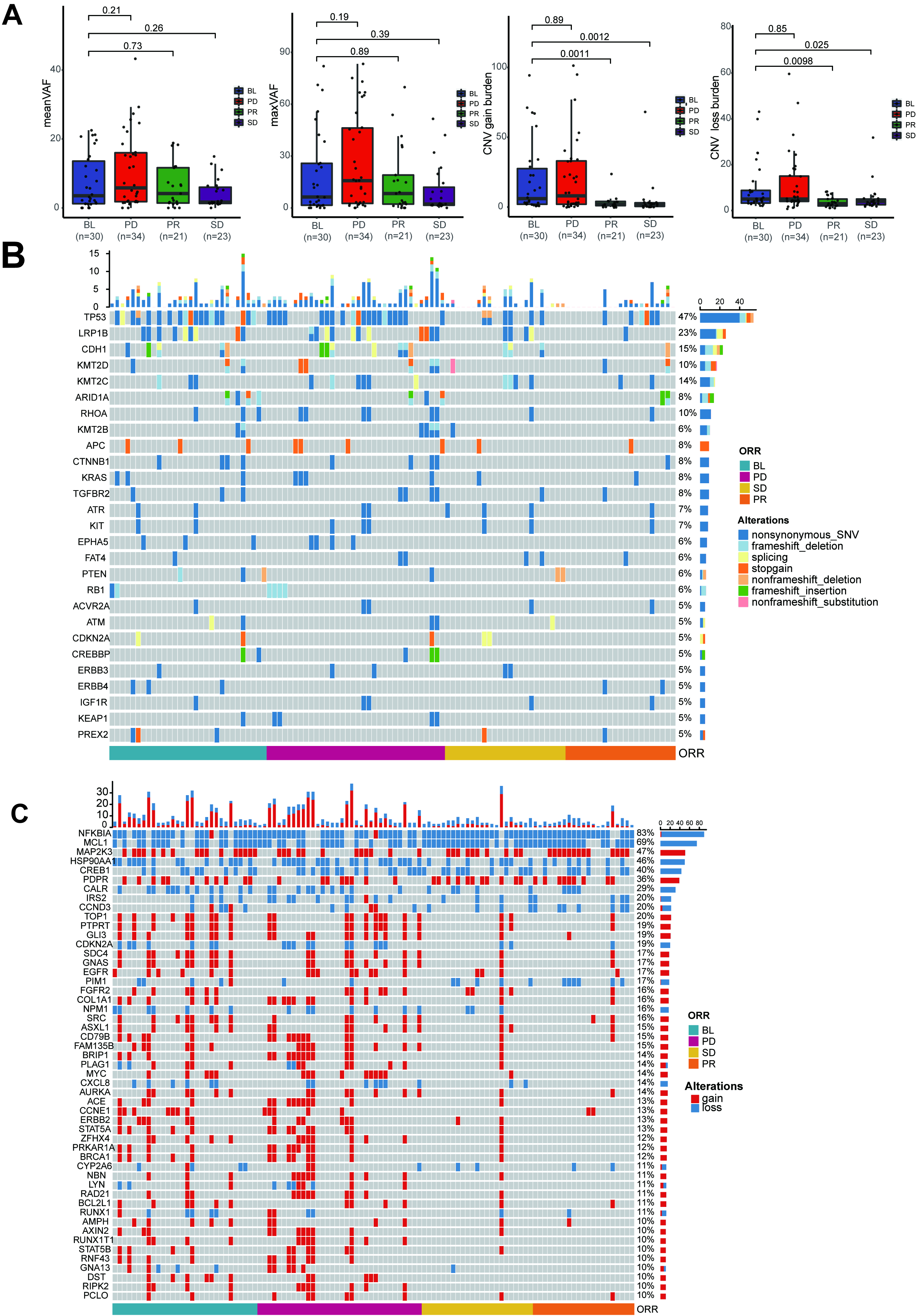

Supplement: Supplementary file 2 — Additional file 2: Figure S2. Dynamic change of genomic features of ctDNA and landscapes of SNVs and CNVs. A: values of meanVAF, maxVAF, CNV gain burden, CNV loss burden at baseline, disease progression and during treatment. B: landscapes of SNVs of all samples. C: landscapes of CNVs of all samples. [file 12967_2022_3567_MOESM2_ESM.tif]

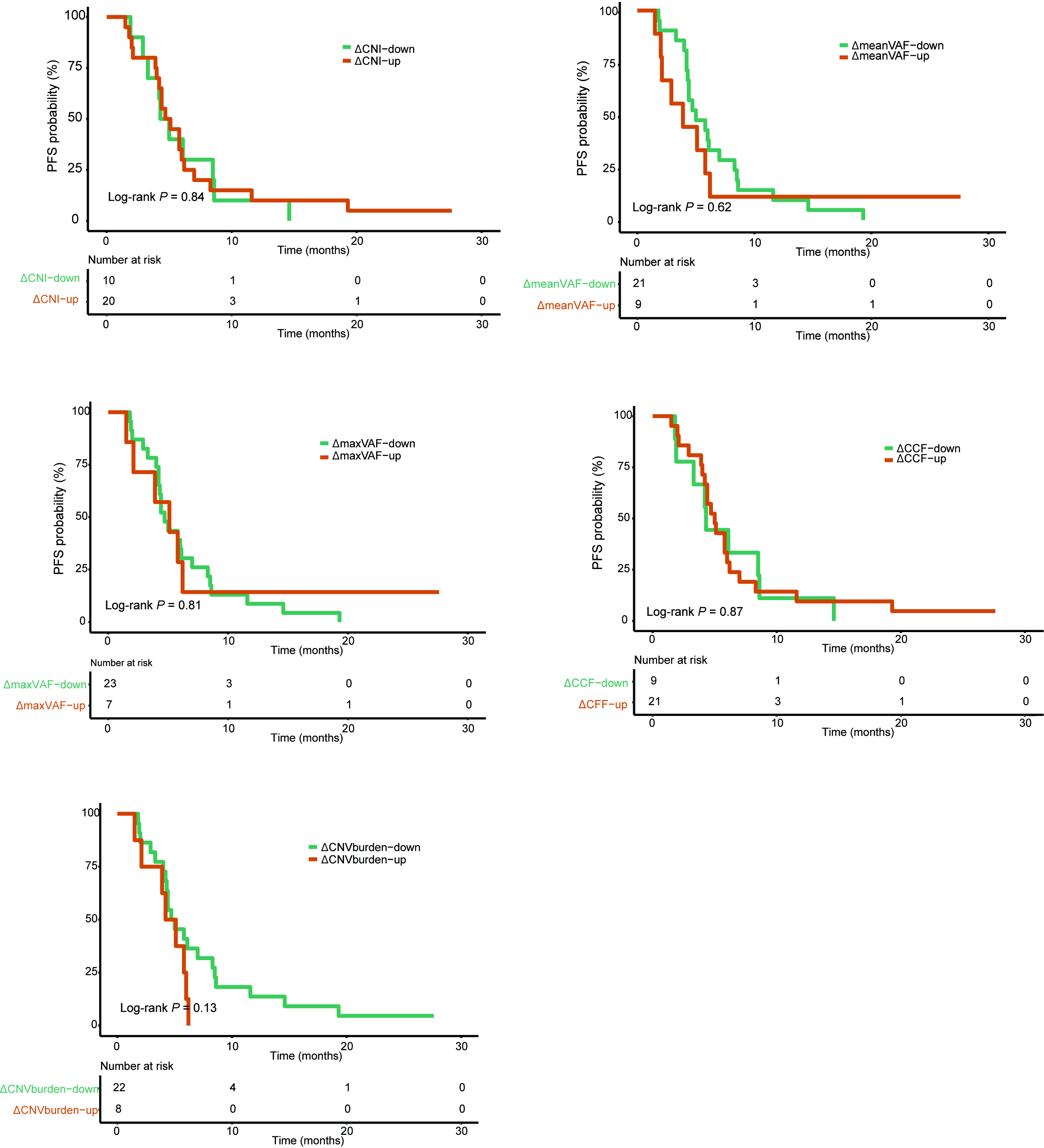

Supplement: Supplementary file 3 — Additional file 3: Figure S3. Correlation between dynamic changes of genomic features of ctDNA and progression free survival of first-line therapy. [file 12967_2022_3567_MOESM3_ESM.tif]

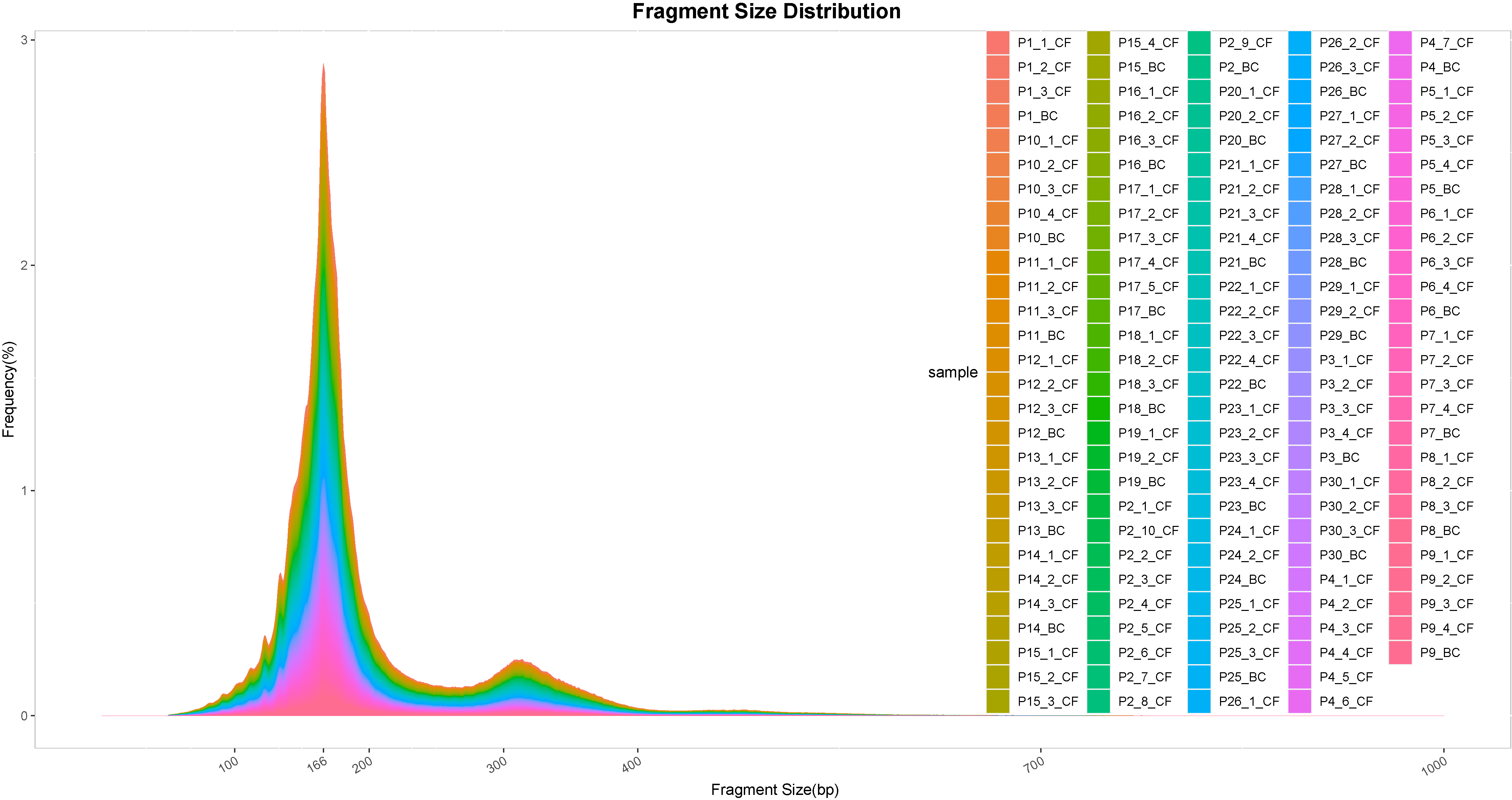

Supplement: Supplementary file 4 — Additional file 4: Figure S4. Insert size distribution. [file 12967_2022_3567_MOESM4_ESM.tif]

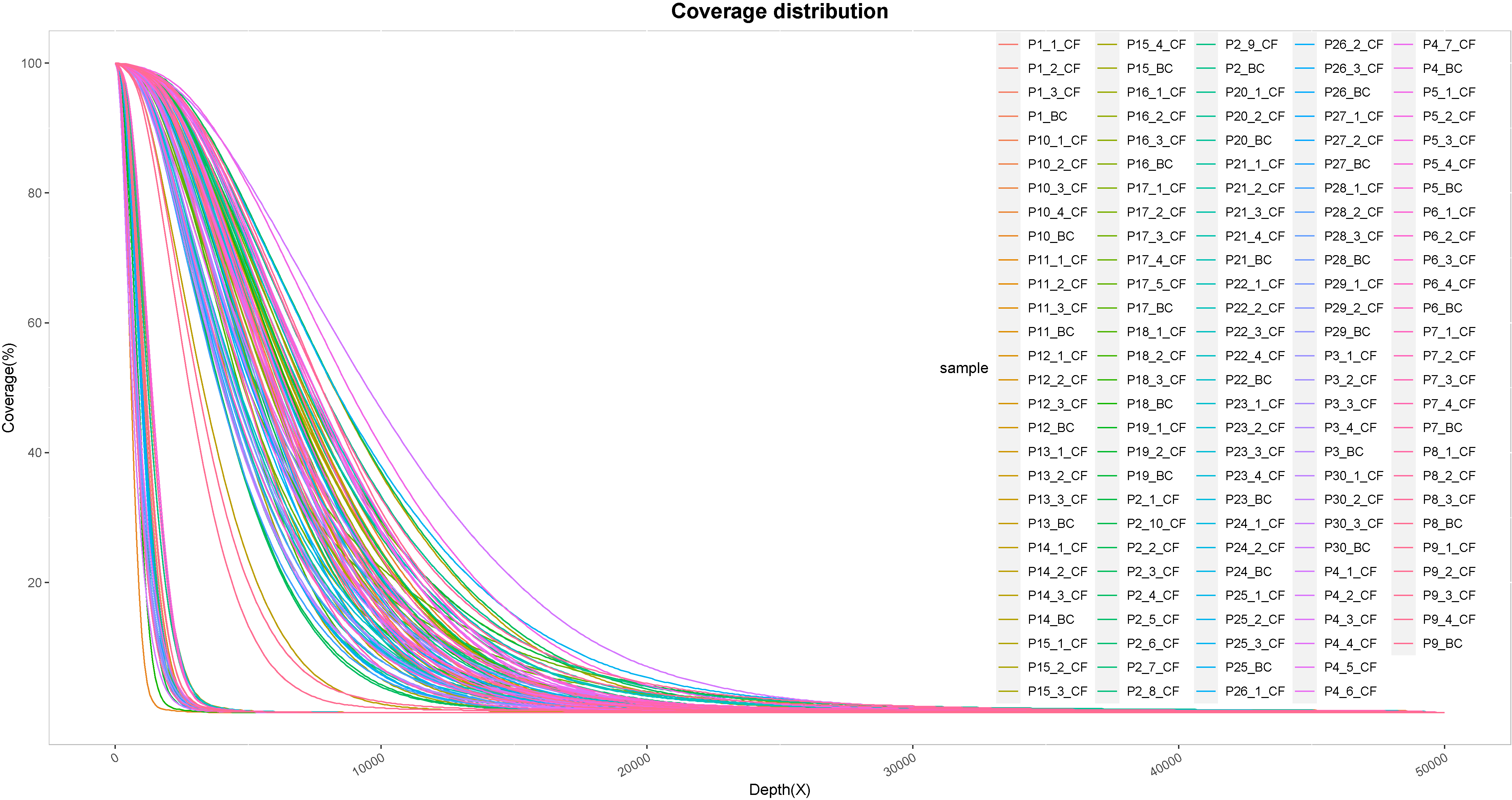

Supplement: Supplementary file 5 — Additional file 5: Figure S5. Coverage distribution. [file 12967_2022_3567_MOESM5_ESM.tif]

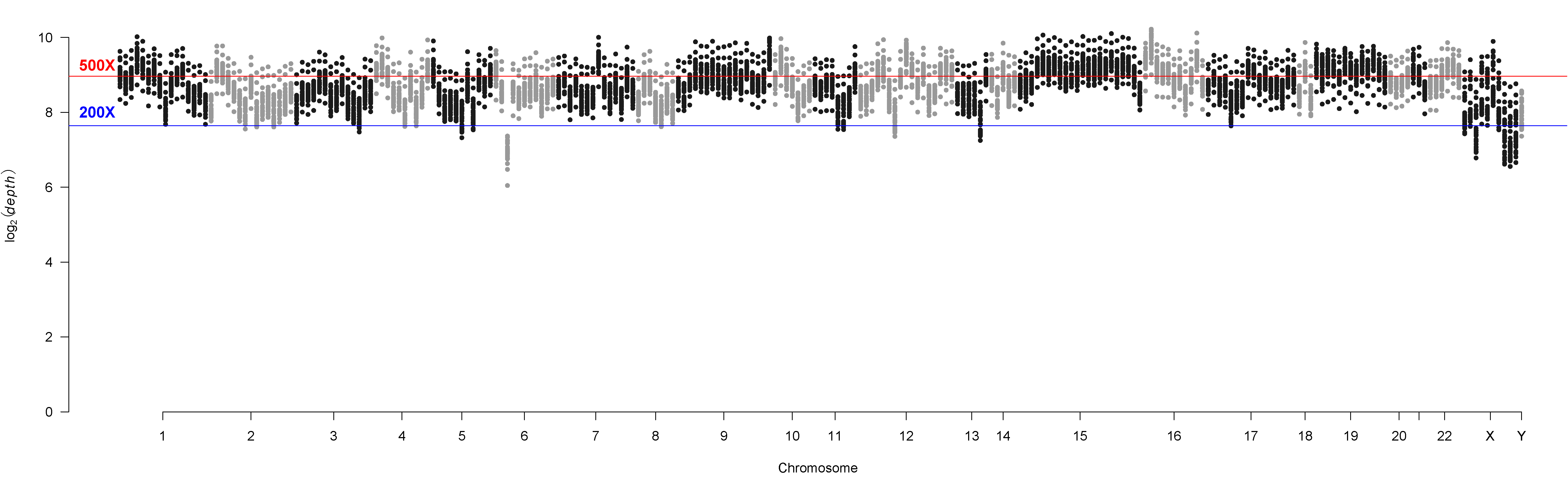

Supplement: Supplementary file 6 — Additional file 6: Figure S6. All uniform Manhattan of blood control samples. [file 12967_2022_3567_MOESM6_ESM.tif]

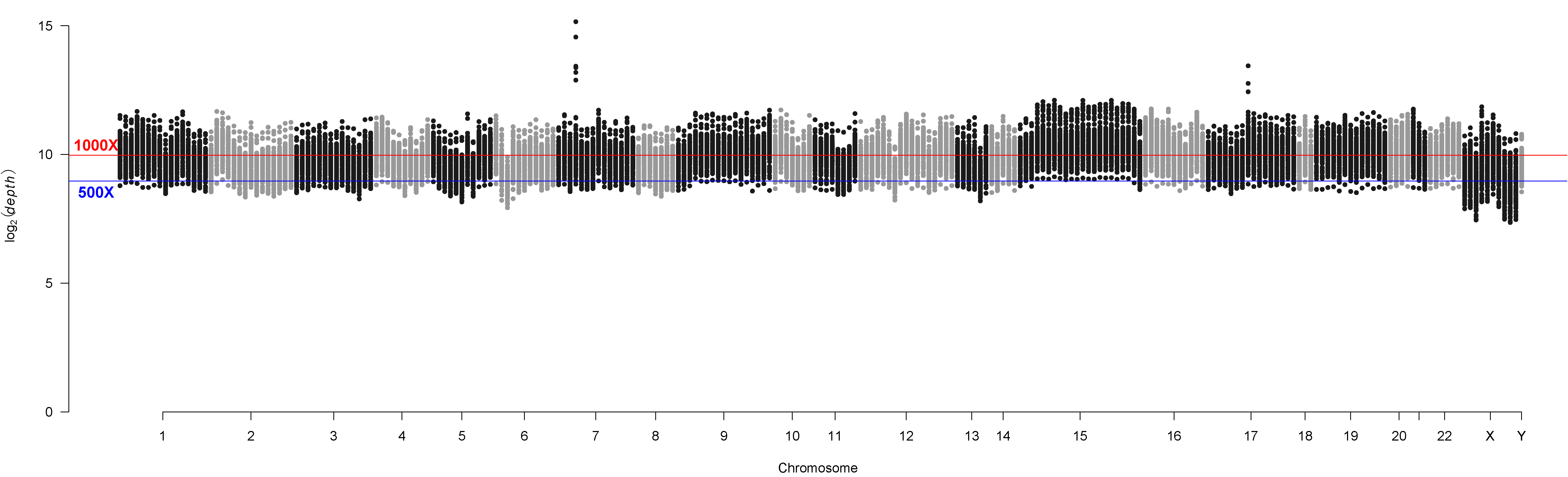

Supplement: Supplementary file 7 — Additional file 7: Figure S7. All uniform Manhattan of cell-free samples [file 12967_2022_3567_MOESM7_ESM.tif]
