# Supplementary material for: Molecular evolutionary process of advanced gastric cancer during sequential chemotherapy detected by circulating tumor DNA
Source: J Transl Med. 2022 Aug 12;20:365. doi: 10.1186/s12967-022-03567-5 (PMC9373478; doi:10.1186/s12967-022-03567-5)
Supplement: Supplementary file 8 — Additional file 8: Table S1. Treatment paradigms of 30 gastric cancer and the corresponding clinical outcomes. Table S2. Examination of the differences between the clinical characteristics of patients in NR and R groups at first-line treatment. [file 12967_2022_3567_MOESM8_ESM.docx]

**Table S1 Clinical information of 30 AGC patients**

| Case | Gender | Age | 1^st^-line | OR^*^ | PFS1 | 2^nd^-line | OR^*^ | PFS2 | Survival | OS |
| --- | --- | --- | --- | --- | --- | --- | --- | --- | --- | --- |
| P1 | M | 72 | FLOT | SD | 27.6 |  |  |  | Y | 27.6 |
| P2 | M | 73 | FLOT | PR | 14.6 |  |  |  | N | 26.6 |
| P3 | F | 60 | SOX | PR | 11.6 |  |  |  | N | 25.5 |
| P4 | F | 68 | SOX | PR | 8.6 | PTX | SD | 4.4 | N | 16.4 |
| P5 | M | 69 | SOX | PR | 8.5 |  |  |  | N | 9.6 |
| P6 | F | 66 | SOX | PR | 8.3 |  |  |  | N | 9.6 |
| P7 | F | 39 | DOS | PR | 7.0 | CPT-11+ Raltitrexed | PR | 3.3 | N | 12.1 |
| P8 | M | 56 | SOX | PR | 6.2 | PX | PD | 1.5 | N | 8.2 |
| P9 | F | 42 | CAPOX | PR | 6.1 | PTX | SD | 2 | Y | 11.6 |
| P10 | F | 64 | DOS | SD | 6.0 |  |  |  | N | 6.7 |
| P11 | M | 77 | SOX | SD | 5.8 |  |  |  | N | 6.2 |
| P12 | M | 56 | FLOT | SD | 5.8 |  |  |  | N | 10.6 |
| P13 | M | 71 | FLOT | PR | 5.0 |  |  |  | N | 8.5 |
| P14 | M | 67 | FOLFOX | PR | 4.7 |  |  |  | Y | 12.1 |
| P15 | M | 69 | FLOT | PR | 4.4 | CPT-11+ Raltitrexed | PD | 0.5 | N | 5.4 |
| P16 | M | 67 | FLOT | PR | 4.3 |  |  |  | N | 12.7 |
| P17 | M | 42 | DOS | PR | 4.2 | FOLFIRI | SD | 6.4 | N | 13.5 |
| P18 | F | 63 | SOX | SD | 4.2 |  |  |  | N | 6.6 |
| P19 | M | 61 | FLOT | SD | 4.0 |  |  |  | N | 6.5 |
| P20 | M | 70 | CAPOX | SD | 3.9 |  |  |  | N | 6.8 |
| P21 | M | 48 | SOX | PR | 3.3 | Nab-PTX | PD | 1.6 | N | 7.6 |
| P22 | F | 37 | FLOT | SD | 2.9 | CPT-11+ Raltitrexed | PD | 1.0 | N | 4.6 |
| P23 | M | 67 | SOX | PD | 2.1 | PX | PR | 8.3 | N | 13.6 |
| P24 | F | 60 | FLOT | PD | 2.0 |  |  |  | N | 7.4 |
| P25 | M | 65 | FLOT | PD | 1.9 | CPT-11+ Raltitrexed | PD | 2.0 | N | 7.9 |
| P26 | M | 68 | FLOT | PD | 1.8 | CPT-11 | PD | 0.7 | N | 3.4 |
| P27 | M | 63 | SOX | PD | 1.5 |  |  |  | N | 2.8 |
| P28 | F | 42 | CAPOX | PR | 19.3 |  |  |  | Y | 28.6 |
| P29 | M | 63 | SOX | SD | 4.4 |  |  |  | N | 13.0 |
| P30 | M | 66 | SOX | SD | 5.1 |  |  |  | N | 9.3 |
| *OR: best objective response assessed during treatment.  PR: partial response; PD: progressive disease; SD: stable disease; Y: yes; N: no. | | | | | | | | | | |

AGC: advanced gastric cancer

**Table S2 Clinical characteristics between patients of NR and R groups at first-line treatment**

| Clinical characteristics | NR | R | *P* value |
| --- | --- | --- | --- |
|  | N=15 | N=15 |  |
| Age | 63.5 (8.93) | 58.6 (12.5) | 0.232 |
| Gender |  |  | 0.699 |
| Female | 4 (26.7%) | 6 (40.0%) |  |
| Male | 11 (73.3%) | 9 (60.0%) |  |
| Hematogenous metastasis |  |  | 0.461 |
| NO | 8 (53.3%) | 5 (33.3%) |  |
| YES | 7 (46.7%) | 10 (66.7%) |  |
| First-line regimens |  |  | 0.682 |
| CAPOX | 1 (6.67%) | 2 (13.3%) |  |
| DOS | 1 (6.67%) | 2 (13.3%) |  |
| FLOT | 7 (46.7%) | 4 (26.7%) |  |
| FOLFOX | 0 (0.00%) | 1 (6.67%) |  |
| SOX | 6 (40.0%) | 6 (40.0%) |  |

R: responsive; NR: non-responsive.
